# Effects of the European Propolis Administration on the Growth Performance, Health Status, and Selected Blood Variables of Calves

**DOI:** 10.1155/vmi/7493364

**Published:** 2025-08-05

**Authors:** Fatemeh Ahmadi, Pavol Mudron, Mehrdad Mohri, Petra Ivancova, Csilla Tothova, Saba Ahmadi, Pavel Gomulec, Jozef Kremen, Simona Mekkova, Kadasi Maryan, Michal Dolnik

**Affiliations:** ^1^Department of Clinical Sciences, Faculty of Veterinary Medicine, Ferdowsi University of Mashhad, Mashhad, Iran; ^2^Clinic of Ruminants, University of Veterinary Medicine and Pharmacy in Košice, Komenského 73, Košice 04181, Slovakia; ^3^Department of General Competencies, University of Veterinary Medicine and Pharmacy in Košice, Komenského 73, Košice 04181, Slovakia

**Keywords:** blood variables, health status, preweaned calves, propolis

## Abstract

**Background:** The effects of propolis supplementation on the performance of dairy calves are not well established. This study aimed to investigate the effects of oral propolis supplementation on the health status, growth performance variables, hematological parameters, and serum biochemical parameters of neonatal dairy calves.

**Methods:** Twenty-four calves were enrolled in this study from 48 h after birth until 7 days of age. A complete clinical examination was performed daily by the same examiner. Blood sampling, body measurements, and lung ultrasonography were conducted on Days 0, 7, 14, and 28. The concentrations of albumin, total protein, total immunoglobulins, γ-globulins, triglycerides, total cholesterol, urea, creatinine, inorganic phosphate, and the activities of aspartate aminotransferase, alanine aminotransferase, and gamma-glutamyl transferase were measured.

**Results:** Propolis supplementation had no significant effect on body weight, average daily gain, length, or height (*p* > 0.05). Pulmonary ultrasonography scores for subclinical pneumonia showed significant variation over time within each group (*p* < 0.05); however, no significant differences were observed between groups. Propolis supplementation significantly reduced the number of days with omphalitis in neonatal calves, likely due to its known anti-inflammatory and antimicrobial effects, although further studies are needed to clarify the underlying mechanisms (*p*=0.016). There were no significant differences in the number of days with fever or diarrhea between groups (*p*=0.44 and 0.15, respectively). No significant differences were found in blood variables between groups. Our results suggest that propolis supplementation did not positively affect growth performance or blood variables.

**Conclusion:** Supplementation with propolis may be beneficial in reducing the incidence of omphalitis in neonatal dairy calves. Nevertheless, further studies are required to fully elucidate the effects of propolis.

## 1. Introduction

Owing to the increase in the world's population and the need to produce more food, the number of livestock farms is increasing daily. Intensive calf-rearing systems are associated with more problems related to neonatal health and a greater risk of neonatal death [1]. The first weeks after the birth of calves are critical for dairy producers because diseases of newborns can overwhelm the immunogenic defense barrier of colostrum against pathogens [2]. Therefore, most producers use antibiotics to reduce the mortality rate caused by diseases such as diarrhea, septicemia, and respiratory diseases during this period of life [3, 4].

Antibiotics have many side effects, and their excessive consumption may cause drug resistance; thus, the food industry is looking for new substances to replace antibiotics that are effective in improving the health of neonates and preventing microbial diseases [5]. Propolis is a suitable alternative that has been used as a traditional substance since ancient times; today, new studies are being conducted on its compounds and therapeutic effects [6]. Between the 17th and 20th centuries, propolis became very famous in European countries for its antibacterial properties. Many researchers have introduced propolis as a powerful natural weapon against pathogenic microorganisms [7]. The antibacterial and immunomodulatory properties of propolis and its potential effects on reducing antibiotic consumption in neonatal dairy calf farms triggered the present study [8]. The word propolis is of Greek origin and consists of two parts: Pro means support or endorsement of something, and Polis means city; so, the combination means support of the city [9].

Propolis is a substance collected by worker bees from the leaf buds of various tree species. Bees have lived on the planet for more than 125 million years, and their ability to fight biological enemies is very sophisticated. Propolis is the most critical “chemical weapon” produced by honey bees and has two unique functions in the structure and defense of the hive. Propolis has a resinous composition, which makes it a special adhesive for plugging holes and cracks, repairing combs, and lining thin comb edges; hence, it is referred to as bee glue [10]. Many of its properties—such as antibacterial, antifungal, antiviral, cytotoxic, antioxidant, anti-inflammatory, and immunomodulatory effects—have been revealed through intensive studies over the last 50 years.

More than 180 chemical constituents have been isolated from propolis, which mainly consists of polyphenols [9]. The major polyphenols are flavonoids, accompanied by phenolic acids and esters, phenolic aldehydes, ketones, and others. The composition of propolis is directly related to the types of tree species in each geographical area, so despite the various studies conducted in recent years on the components of propolis, new components may be reported based on further studies [11].

Propolis is not a new discovery in medicine and has a long history. The first reports of the use of propolis as a medicine date back to 300 BC, when it was used as a common local remedy throughout the world in both internal and external applications [12]. Numerous studies involving the simultaneous efforts of phytochemists and pharmacologists have concluded that the biological activity and chemistry of different propolis samples are entirely different [13].

Currently, the primary source of European propolis is the resinous exudates of poplar trees, mainly the black poplar Populus nigra, in temperate zones of the Earth. For this reason, European propolis contains typical poplar bud phenolics: flavonoid aglycones (flavones and flavanones), phenolic acids, and their esters [10]. Clinical trials are needed to evaluate propolis supplementation in healthy and sick individuals and to understand the mechanisms of its potential to promote health.

Recent studies have highlighted propolis as a promising natural supplement with multiple beneficial effects on animal health [14]. Propolis exhibits notable antimicrobial, antioxidant, anti-inflammatory, and immunomodulatory properties in various livestock species [15]. In neonatal calves, supplementation with propolis has been reported to enhance immune responses, reduce the incidence of infections, and improve growth performance by modulating blood biochemical parameters and lowering oxidative stress [6]. Moreover, propolis is considered a potential natural alternative to antibiotics, which may help mitigate the rising problem of antimicrobial resistance while supporting calf health during critical early life stages [16, 17]. Despite these promising findings, research specifically focusing on European propolis and its effects on neonatal dairy calves remains limited, underscoring the need for further investigation [10].

The hypothesis of our study was as follows: Does European propolis supplementation have beneficial effects on the blood parameters, health status, and growth performance of neonatal dairy calves?

The objective of this study was to evaluate the effects of oral European propolis supplementation on the health status, growth performance, and blood variables of neonatal dairy calves.

## 2. Materials and Methods

### 2.1. Propolis Extract

Raw propolis was supplied by beekeepers of Kosice and nearby regions in August 2022. Kosice is a city in the Republic of Slovakia with a moderately continental climate. A total of 500 g of propolis was collected by scraping the inside of the boxes of *Apis mellifera* bees by a beekeeper. According to a previous study, the collected propolis was stored at 4°C for less than 1 week before extraction [18].

The propolis extraction was performed according to Alencar et al. [19], with minor modifications to improve extraction efficiency, which may be related to the source and quality of the propolis. The propolis was ground mechanically using a mortar and pestle to crush it into a fine powder. After grinding, 100 g of propolis powder was weighed using a digital scale (Ohaus, Switzerland). Then, 450 mL of 80% ethanol was mixed with the propolis powder. The suspension was placed on a magnetic stirrer (Heidolph, Germany) at 200 rpm for 24 h at room temperature. The prepared suspension was left standing for 3 h to allow two distinct phases to form, then filtered through Whatman paper No. 4. To evaporate the alcohol from the precipitate, a drying machine (Chirana, Czechoslovakia) was used. Finally, the weight of the remaining sediment was measured, and 35 g were discarded. The remaining alcoholic extract contained 65 g of propolis from the original 100 g. Therefore, 65 g of propolis was added to 400 mL of solvent, resulting in a 16.25% concentration. To create a tincture with a concentration of 32.5%, the solution was placed in a 70°C water bath (Memmert, Germany) until the final volume was halved.

To prepare a placebo, the ethanol concentration was determined using an alcoholometer. The final alcohol concentration was 46%. The control group design accounted for the ethanolic content of the treatment group: Group 1 received 4 mL of 32.5% propolis ethanolic extract (PEE), while Group 2 received 4 mL of 46% ethanol. This ensured that both groups were exposed to similar ethanol levels, isolating the effect of propolis itself. Ethanol in the control group served to control for any potential solvent effects.

### 2.2. Animal Feeding and Treatments

This study was conducted on a 400-head dairy cattle farm affiliated with the University of Veterinary Medicine and Pharmacy of Kosice, located in Zemplínska Teplica near Kosice, during the period from September 6 to October 18, 2022, when the average temperature, humidity, and rainfall were 14.5°C, 19.22%, and 105 mm, respectively.

Twenty-four neonatal dairy calves (Holstein and Holstein crosses) were included from 48 h after birth until 7 days of age. Each calf was free from failure of passive transfer (FPT), confirmed by sufficient serum total protein concentrations. To detect FPT, TP and globulin levels were measured, and a sodium sulfate turbidity test was conducted 48 h after birth, prior to enrollment. There was no significant difference in initial weight between groups; both had an average starting weight of approximately 45.3 kg. Each group contained six male and six female calves.

The experimental calves were housed individually and identified by neck numbers. Prior to inclusion, clinical evaluations were performed, including a physical exam, total protein measurement, and lung ultrasonography to rule out pneumonia.

### 2.3. Housing Conditions

Calves were separated from dams immediately after birth and received 6 L of pooled colostrum within the first 24 h. They were fed commercial milk replacer twice daily, with calf starter and water available ad libitum. Individual shelters had straw bedding and dimensions of 300 cm (length) × 145 cm (roof area) × 120 cm (height) × 110 cm (width), separated by iron walls.

Group 1 received 4 mL of 32.5% (325 mg/mL) PEE for 14 consecutive days; Group 2 received 4 mL of 46% ethanol (placebo) for 14 consecutive days. Both treatments were mixed into the evening milk feeding to ensure full intake and minimize stress during morning clinical procedures.

### 2.4. Weight Estimation

Weight was estimated using a girth tape by measuring the body circumference behind the elbow joint with the calf in a natural stance. Initial weight was recorded before the trial began and again on Days 7, 14, 21, and 28 using the same method.

Although heart girth is a practical field method for weight estimation, it may lack precision compared to digital scales. Girth tape accuracy can vary depending on calf posture and operator consistency, especially in neonates [20].

### 2.5. Morphometric Measurements

Body length (from shoulder point to ischium) and height (from withers to floor) were measured on Days 0, 7, 14, 21, and 28. All measurements were taken by the same person between 10:00 and 12:00 a.m. to minimize observer variability.

### 2.6. Blood Sampling

Blood samples were collected from the jugular vein between 10:00 and 12:00 a.m. on Days 0, 7, 14, and 21. Samples were transferred to plain tubes for serum separation and sodium sulfate turbidity testing.

Anticoagulated blood was refrigerated at 4°C and transported to the lab within 2 h. Hematological variables were measured using a cell counter (Mindray BC 2800Vet, China). Serum was separated by centrifugation at 4000 rpm (Hettich Universal 320/320R, Germany) for 10 min. Total immunoglobulin was measured using the zinc sulfate turbidity test [21], and serum samples were stored at −20°C until analysis.

To monitor animal health and potential PEE side effects, serum biochemical variables were measured: albumin (Alb), total protein (TP), triglyceride (TG), total cholesterol (TChol), urea, creatinine (Cre), inorganic phosphate (Pi), and enzyme activities (AST, ALT, GGT), using commercial kits (Randox, UK) on an Alize autoanalyzer (Lisabio, France). Calcium was measured via atomic absorption spectrometry (PerkinElmer, USA).

Serum protein fractions were separated using zone electrophoresis on agarose gel (pH 8.8) with an automated Hydrasys system (Sebia, France), following the Hydragel 7 Proteine kit protocol. Ten microliters of each sample were applied to numbered wells. Control serum (Human Normal, Sebia) was included. Electrophoresis was performed for 15 min at 20°C (10 W, 40 mA, 240 V). Gels were stained with amidoblack, destained, and dried. A densitometer (Epson Perfection V700, USA) scanned the gels, and protein fractions were analyzed using Phoresis v5.50 software (Sebia), with manual verification if needed.

Fractions included: albumin, alpha-1 (α1), alpha-2 (α2), beta-1 (β1), beta-2 (β2), and gamma (γ)-globulins. Relative concentrations (%) were based on optical absorbance; absolute concentrations (g/L) were calculated using total protein values.

### 2.7. Lung Ultrasonography

**Figure 1:**
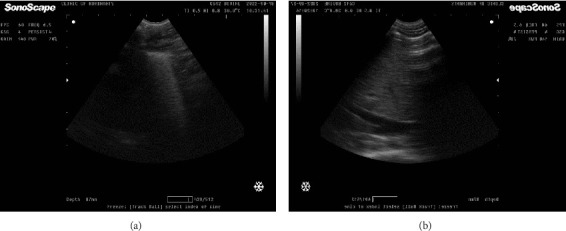
Spleen (a) and liver with lung (b) images are hallmarks of left- and right-sided lung imaging.

Concurrent with blood sampling, lung ultrasonography was performed on both sides of the thorax using a SonoScape ultrasound machine (Shenzhen, China) (Figure 1).

### 2.8. Lung Ultrasonography

The thorax of each calf was symmetrically scanned from caudal to cranial between the 10th and 3rd intercostal spaces using a 7.7 MHz convex probe, which was directly applied to the thorax after 70% ethylic alcohol was sprayed onto the area of interest to achieve good image quality without clipping the area.

The rapid scoring system designed by Adams and Buczinski in 2016 for lung injuries was used to interpret the ultrasound images [22]. The ultrasonography scores of the calves' lungs were recorded on Days 0, 7, 14, and 28.

### 2.9. Examination of Health Status

A complete clinical examination was performed daily by the same person throughout the study. A rectal temperature equal to or greater than 39.5°C was considered indicative of fever, in accordance with established veterinary standards and farm protocol [23]. Fecal consistency was scored using the system described by Larson et al. [24], ranging from 1 to 4; scores of 3 or higher were classified as diarrhea.

Omphalitis was defined as a warm, swollen area associated with the navel [25]. To evaluate the condition, umbilical swelling was measured daily using gentle palpation to assess the diameter at the widest point. A flexible measuring tape was used to record the size of the swelling in centimeters (cm). Measurements were taken carefully, and results were recorded to track changes over time. In line with previous studies [26], any umbilical diameter greater than 2 cm was considered indicative of omphalitis. This threshold is consistent with the criteria outlined in the referenced literature. Additionally, associated symptoms such as fever and the consistency of the swelling were monitored and recorded for comparison during the follow-up period. These findings will be incorporated into the revised manuscript to provide a more comprehensive analysis of omphalitis in the context of our study.

### 2.10. Statistical Analysis

Statistical analysis was performed using SPSS version 16 software (IBM, SPSS, Inc., USA). The distribution of the data was evaluated via the Shapiro–Wilk test. For data with a normal distribution at all sampling times, repeated measures analysis of variance (RM ANOVA) was used, and the effects of trial groups, time, and time–group interactions were examined. For non-normally distributed data, appropriate transformations were applied. If normalization was not possible, the Mann–Whitney U test was used for comparisons between trial groups at each sampling time.

As the sample size was not determined in advance due to the exploratory nature of the study, post hoc comparisons were performed in each analysis to obtain precise results. Differences in the duration of diarrhea and omphalitis were evaluated by independent samples t-tests. In all analyses, *p* < 0.05 was considered statistically significant.

### 2.11. Ethics Statement

Approval from the Ethics Commission of the University of Veterinary Medicine and Pharmacy in Kosice was obtained (Protocol No. EKVP/2022-24).

## 3. Results

PEE supplementation had no significant effect on body weight, daily weight gain, length, or height (*p* > 0.05; Table 1).

There was no significant difference in total weight gain (Table 1) or average daily weight gain between the PEE group (mean ± SD = 0.43 ± 0.06 kg/day) and the placebo group (mean ± SD = 0.41 ± 0.05 kg/day).

**Figure 2:**
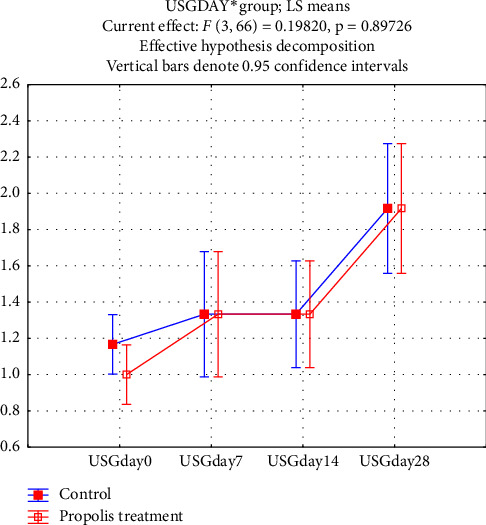
Lung ultrasonography scores on Days 0, 7, 14, and 28.

Pulmonary scores obtained via lung ultrasonography for subclinical pneumonia varied significantly over time, but no significant differences were observed between the treatment and control groups (Figure 2).

During the study, 7 calves in the PEE group and 10 calves in the control group developed omphalitis. The duration of omphalitis was significantly shorter in the propolis-treated calves compared to controls (mean ± SD: 2.66 ± 2.60 days vs. 6.66 ± 4.65 days, respectively; *p*=0.016).

The average values for key health parameters (including clinical signs) for calves in both trial groups at the end of weeks 1, 2, 3, and 4 are presented in Table 2.

Values represent means ± standard error (SE). Variables include rectal temperature (°C), heart rate (beats per minute), and respiratory rate (breaths per minute). Statistical analysis was performed using repeated measures ANOVA to evaluate the effects of treatment group, time, and the interaction between group and time (Group × Time). *p*-Values for group, time, and interaction effects are provided. Statistical significance was defined as *p* < 0.05.

Eight calves in the PEE group and nine calves in the control group developed diarrhea during the study; however, there was no significant difference in the number of days with diarrhea between the groups (*p*=0.15).

The median number of treatment days for diarrhea was 4.0 days in the PEE group and 3.5 days in the control group, with no significant difference between them.

There was also no significant difference in hematological variables between the trial groups (Table 3).

The serum biochemical variables showed no significant differences between the PEE and placebo groups throughout the study (Table 4).

## 4. Discussion

The present study aimed to investigate the effects of oral European propolis supplementation on the health status and growth performance of neonatal dairy calves. Although European propolis extract has a bitter taste, all calves readily consumed the supplemented milk without refusal. Consequently, milk intake remained similar between the PEE and placebo groups throughout the study period.

In this study, propolis significantly reduced neonatal omphalitis (*p*=0.016). Propolis has been shown to have antimicrobial properties, which may contribute to its effectiveness in reducing omphalitis in neonatal calves. Omphalitis is an inflammation of umbilical structures that may involve the umbilical arteries, vein, urachus, or adjacent tissues. Omphalophlebitis can extend into the liver and cause hepatic abscessation [27].

Given that the primary cause of omphalitis is inflammation, the authors believe that propolis may exhibit anti-inflammatory properties. As inflammation is a common issue in neonatal calves, further studies are warranted to explore the anti-inflammatory effects of propolis in this context.

We used a 32.5% (325 mg/mL) ethanolic extract of propolis, selected based on previous research and pilot data supporting its efficacy [28]. While different concentrations may yield different results, this study focused on a single, standardized dose. Future research should assess a broader range of concentrations and dosing regimens.

There were no significant differences in the number of days with fever or diarrhea between groups. In contrast, studies by Kabiloglu et al. [29], Slanzon et al. [28], and Yucel et al. [30] reported reduced diarrhea incidence after PEE supplementation for more than 30 days. The discrepancy among findings may stem from differences in propolis composition [31], pathogen types, and geographic origin. Propolis from the Middle East shows higher antimicrobial activity against a broad spectrum of bacteria than that from Ireland or Germany [8]. Given the Slovak origin of our propolis, geographic proximity to Germany may have contributed to its lower efficacy.

Digital scales are the gold standard for body weight measurement, but due to field conditions and animal stress concerns, we used a calibrated heart girth tape. This method has shown strong correlation with body weight in neonatal calves when used consistently [20, 32].

Although Tolon et al. [33] observed increased weight gain in female calves treated with propolis, our study showed no effect on growth performance (*p* > 0.05, Table 1), which aligns with Slanzon et al. [28] and Morsy et al. [34]. In contrast, Yaghoubi et al. [35] reported higher body weights in calves receiving flavonoid-rich propolis extracts. The higher concentration of bioactive compounds in purified flavonoids, versus crude PEE used in our study, may explain the difference.

Kupczyński et al. [36] found increased daily gain in calves supplemented with propolis, which may have been influenced by mild diarrhea in the control group. Other influencing factors include seasonality, flora near the hive, altitude, daylight exposure [37], and extraction methods [4, 38].

Bovine respiratory disease (BRD) remains a major clinical issue. Lung ultrasonography is superior to auscultation for detecting subclinical pneumonia [39, 40]. Our findings confirmed its value: while there was no difference between treatment groups, significant time-based variation in ultrasonography scores within groups was noted (Figure 2). These likely reflect maturation of immune responses and lung development [41–43].

The risk of diarrhea peaks during the first week, while respiratory diseases rise around week 4 [25, 43]. Our findings aligned with this timeline, confirming dynamic health risks in early calfhood.

Previous work has evaluated propolis as a natural remedy for respiratory disease prevention [44]. However, the dose or duration used here may have been insufficient. Further studies should explore higher or prolonged doses and administration routes.

Regarding hematological parameters, our results showed no significant group differences, consistent with Prado-Calixto et al. [45] and Kupczyński et al. [36]. Nonetheless, propolis may benefit erythropoiesis or iron metabolism, even without altering complete blood count values.

Age-related variation in blood parameters may have influenced results [46]. The lack of significant impact on total protein, albumin, or globulins was also consistent with Slanzon et al. [28]. Earlier or more frequent blood sampling might better detect transient immunoglobulin changes during passive transfer.

Morsy et al. [34] found that propolis modulates immunoglobulin levels. We did not observe this effect, likely due to differences in formulation, dosage, and timing.

No significant group differences in urea, creatinine, or triglycerides were observed, consistent with Morsy et al. [34]. Although propolis is sometimes reported to have hepatoprotective effects, no changes in liver enzyme activity were noted in our study, likely due to consistent management and housing across groups. Finally, calcium and phosphorus levels were unaffected, as also noted by Kupczynski et al. [36].

## 5. Conclusion

The results of this study suggest that European propolis supplementation had no significant positive effects on growth performance, hematological indices, or serum biochemical parameters in neonatal dairy calves. However, it may help reduce the incidence of omphalitis, potentially lowering the need for antibiotic therapy in intensive farming systems.

Further studies should assess different durations, doses, and methods of administration to fully elucidate the effects of propolis on calf health and development.

## Figures and Tables

**Table 1:** Performance of calves supplemented with PEE or placebo during the study.

Variable	PEE		Control		Group	Time	Time ^∗^ group
	Mean	Std. error	Mean	Std. error			
Total gain (kg)	11.5	1.56	12.16	1.85	0.79	—	—
Weekly weight (kg)	49.125	1.25	49.20	1.25	0.963	0.000	0.835
Daily gain per week (kg)	0.548	0.081	0.580	0.081	0.782	0.000	0.777
Height (cm)	79.29	1.008	80.854	1.008	0.285	0.000	0.446
Length (cm)	74.10	1.034	75.68	1.034	0.290	0.000	0.171

*Note:* Std. error = standard error; Time ^∗^ group = interaction between time and group.

Abbreviation: PEE = propolis ethanolic extract.

^∗^PEE: 4 mL of 32.5% (325 mg/mL) propolis tincture in milk; placebo: 4 mL of 46% alcohol in milk.

**Table 2:** Health characteristics of calves in the PEE and placebo groups measured at multiple timepoints during the study.

Variable	PEE		Control		Group	Time	Time ^∗^ group
	Mean	Std. error	Mean	Std. error			
Temperature (°C)	38.64	0.2	38.59	0.2	0.15	0.37	0.3
Heart rate (beats/min)	113.65	3.95	122.12	3.95	0.14	0.0	0.42
Respiratory rate (breaths/min)	33.93	1.29	33.99	1.29	0.97	0.0	0.53

*Note:* Std. error = standard error; Time ^∗^ group = interaction between time and group.

Abbreviation: PEE = propolis ethanolic extract.

**Table 3:** Hematological parameters of calves in the PEE and placebo groups.

Variable	PEE		Control		Group	Time	Time ^∗^ group
	Mean	Std. error	Mean	Std. error			
RBC (10^9^/L)	7.59	0.37	7.9	0.39	0.57	0.052	0.55
Hb (g/dL)	10.44	0.59	10.22	0.61	0.8	0.000	0.96
HTCT (%)	0.27	0.016	0.28	0.017	0.81	0.039	0.46
MCV (fL)	35.49	0.65	34.68	0.68	0.4	0.000	0.172
WBC (× 10^3^/μL)	10.77	1.52	12.24	1.59	0.39	0.000	0.87

*Note:* RBC = red blood cells (10^9^/L); Hb = hemoglobin (g/dL); HTCT = hematocrit (%); MCV = mean corpuscular volume (fL); WBC = white blood cells (× 10^3^/μL); Std. error = standard error; Time ^∗^ group = interaction between time and group.

**Table 4:** Serum biochemical variables of calves in the PEE and placebo groups.

Variable	PEE		Control		Group	Time	Time ^∗^ group
	Mean	Std. error	Mean	Std. error			
TP (g/L)	58.94	1.31	58.51	1.31	0.81	0.000	0.94
Alb (g/L)	33.17	0.39	33.58	0.39	0.46	0.000	0.4
TIg (U zst^1^)	15.77	1.11	15.91	1.11	0.93	0.007	0.76
γ globulins (g/L)	7.30	0.75	6.84	0.75	0.66	0.000	0.55
AST (Ukat/L)	0.81	0.058	0.8	0.058	0.89	0.015	0.81
ALT (Ukat/L)	6.65	0.66	6.43	0.66	0.81	0.000	0.39
GGT (Ukat/L)	3.18	1.12	2.87	1.12	0.85	0.012	0.89
TG (mmol/L)	0.47	0.037	0.46	0.037	0.93	0.000	0.75
Tchol (mmol/L)	2.49	0.16	2.59	0.16	0.65	0.000	0.28
Urea (mmol/L)	5.31	0.38	5.01	0.38	0.58	0.025	0.6
Cre (μmol/L)	90.61	3.33	90.23	3.33	0.94	0.61	0.11
Ca (mmol/L)	2.63	0.02	2.59	0.02	0.23	0.005	0.79
P (mmol/L)	2.49	0.054	2.42	0.054	0.39	0.197	0.135

*Note:* TP = total protein (g/L); Alb = albumin (g/L); TIg = total immunoglobulins (U ZST1); γ globulins = gamma globulins (g/L); AST = aspartate aminotransferase (Ukat/L); ALT = alanine aminotransferase (Ukat/L); GGT = gamma-glutamyl transferase (Ukat/L); TG = triglycerides (mmol/L); Tchol = total cholesterol (mmol/L); Urea = urea (mmol/L); Cre = creatinine (μmol/L); Ca = calcium (mmol/L); P = phosphorus (mmol/L); Std. error = standard error; Time ^∗^ group = interaction between time and group.

^1^Units of the zinc sulfate test.

## Data Availability

The data that support the findings of this study are available from the corresponding author upon reasonable request.

## References

[1] McCorquodale C. E., Sewalem A., Miglior F. (2013). Short Communication: Analysis of Health and Survival in a Population of Ontario Holstein Heifer Calves. *Journal of Dairy Science*.

[2] Chase C. C., Hurley D. J., Reber A. J. (2008). Neonatal Immune Development in the Calf and Its Impact on Vaccine Response. *Veterinary Clinics of North America: Food Animal Practice*.

[3] Raymond M. J., Wohrle R. D., Call D. R. (2006). Assessment and Promotion of Judicious Antibiotic Use on Dairy Farms in Washington State. *Journal of Dairy Science*.

[4] Constable P. D. (2003). Use of Antibiotics to Prevent Calf Diarrhea and Septicemia. *Bovine Practitioner*.

[5] Low C. X., Tan L. T., Ab Mutalib N. S. (2021). Unveiling the Impact of Antibiotics and Alternative Methods for Animal Husbandry: A Review. *Antibiotics*.

[6] El-Nagar H. A., El-Hais A. M., Abd El-Aziz A. H. (2023). Growth Performance, Immunity, and General Health Status of Newborn Friesian Calves Fed Milk Supplemented With Propolis, Thyme, or Their Combination, As Antioxidants, During the Suckling Period. *Advances in Animal and Veterinary Sciences*.

[7] Özdemir V., Yanar M., Koçyiğit R. (2022). General Properties of Propolis and Its Usage in Ruminants. *Journal of the Hellenic Veterinary Medical Society*.

[8] Przybyłek I., Karpiński T. M. (2019). Antibacterial Properties of Propolis. *Molecules*.

[9] Castaldo S., Capasso F. (2002). Propolis, An Old Remedy Used in Modern Medicine. *Fitoterapia*.

[10] Bankova V. (2005). Recent Trends and Important Developments in Propolis Research. *Evidence-Based Complementary and Alternative Medicine*.

[11] Bayram N. E., Gerçek Y. C. (2017). Major Constituents of Different Propolis Samples. *Hacettepe Journal of Biology and Chemistry*.

[12] Sforcin J. M. (2007). Propolis and the Immune System: A Review. *Journal of Ethnopharmacology*.

[13] Lustosa S. R., Galindo A. B., Nunes L. C., Randau K. P., Rolim Neto P. J. (2008). Própolis: Atualizações Sobre a Química e a Farmacologia. *Revista Brasileira de Farmacognosia*.

[14] Bankova V., Popova M., Trusheva B. (2014). Propolis volatile Compounds: Chemical Diversity and Biological Activity: A Review. *Chemistry Central Journal*.

[15] Sforcin J. M., Bankova V. (2011). Propolis: Is There a Potential for the Development of New Drugs?. *Journal of Ethnopharmacology*.

[16] Kerek Á., Csanády P., Tuska-Szalay B., Kovács L., Jerzsele Á. (2023). In Vitro Efficacy of Hungarian Propolis Against Bacteria, Yeast, and Trichomonas Gallinae Isolated From pigeons—A Possible Antibiotic Alternative?. *Resources*.

[17] Svetikiene D., Zamokas G., Jokubaite M. (2024). The Comparative Study of the Antioxidant and Antibacterial Effects of Propolis Extracts in Veterinary Medicine. *Veterinary Sciences*.

[18] Park Y. K., Ikegaki M. (1998). Preparation of Water and Ethanolic Extracts of Propolis and Evaluation of the Preparations. *Bioscience, Biotechnology, and Biochemistry*.

[19] Alencar S. M., Oldoni T. L. C., Castro M. L. (2007). Chemical Composition and Biological Activity of a New Type of Brazilian Propolis: Red Propolis. *Journal of Ethnopharmacology*.

[20] Heinrichs A. J., Rogers G. W., Cooper J. B. (1992). Predicting Body Weight and Wither Height in Holstein Heifers Using Body Measurements. *Journal of Dairy Science*.

[21] McEwan A. D., Fisher E. W., Selman I. E., Penhale W. J. (1970). A Turbidity Test for the Estimation of Immune Globulin Levels in Neonatal Calf Serum. *Clinica Chimica Acta*.

[22] Adams E. A., Buczinski S. (2016). Short Communication: Ultrasonographic Assessment of Lung Consolidation Postweaning and Survival to the First Lactation in Dairy Heifers. *Journal of Dairy Science*.

[23] Foster D. M., Smith G. W. (2009). Pathophysiology of Diarrhea in Calves. *Veterinary Clinics of North America: Food Animal Practice*.

[24] Larson L. L., Owen F. G., Albright J. L., Appleman R. D., Lamb R. C., Muller L. D. (1977). Guidelines Toward More Uniformity in Measuring and Reporting Calf Experimental Data. *Journal of Dairy Science*.

[25] Svensson C., Lundborg K., Emanuelson U., Olsson S. O. (2003). Morbidity in Swedish Dairy Calves From Birth to 90 Days of Age and Individual Calf-Level Risk Factors for Infectious Diseases. *Preventive Veterinary Medicine*.

[26] Fahmy M. (2018). *Umbilicus and Umbilical Cord*.

[27] Smith P. B., Van Meter D., Pusterla N. (2021). *Large Animal Internal Medicine*.

[28] Slanzon G. S., Toledo A. F., Silva A. P. (2019). Red Propolis as an Additive for Preweaned Dairy Calves: Effect on Growth Performance, Health, and Selected Blood Parameters. *Journal of Dairy Science*.

[29] Kabiloglu A., Kocabagli N., Kekec A. I. (2023). Effects of Propolis Extract on Growth Performance and Health Condition of Dairy Calves. *Tropical Animal Health and Production*.

[30] Yucel B., Önenç A., Kaya A., Altan Ö. (2015). Effects of Propolis Administration on Growth Performance and Neonatal Diarrhea of Calves. *SOJ Veterinary Sciences*.

[31] Almuhayawi M. S. (2020). Propolis as a Novel Antibacterial Agent. *Saudi Journal of Biological Sciences*.

[32] Sherwin V., Hyde R., Green M., Remnant J., Payne E., Down P. (2021). Accuracy of Heart Girth Tapes in the Estimation of Weights of Pre‐Weaned Calves. *Veterinary Record Open*.

[33] Tolon B., Önenç A., Kaya A., Altan Ö. (2002). Effects of Propolis on Growth of Calves. *1st German Congress for Bee Products and Apitherapy*.

[34] Morsy A. S., Abdalla A. L., Soltan Y. A. (2013). Effect of Brazilian Red Propolis Administration on Hematological, Biochemical Variables and Parasitic Response of Santa Inês Ewes During and After Flushing Period. *Tropical Animal Health and Production*.

[35] Yaghoubi S. M., Ghorbani G. R., Rahmani H. R., Nikkhah A. (2008). Growth, Weaning Performance and Blood Indicators of Humoral Immunity in Holstein Calves Fed Supplemental Flavonoids. *Journal of Animal Physiology and Animal Nutrition*.

[36] Kupczyński R., Adamski M., Falta D., Roman A. (2012). The Efficiency of Propolis in Post-Colostral Dairy Calves. *Archives of Animal Breeding*.

[37] Silva-Carvalho R., Baltazar F., Almeida-Aguiar C. (2015). Propolis: A Complex Natural Product With a Plethora of Biological Activities That Can Be Explored for Drug Development. *Evidence-based Complementary and Alternative Medicine*.

[38] Cui J., Duan X., Ke L. (2022). Extraction, Purification, Structural Character and Biological Properties of Propolis Flavonoids: A Review. *Fitoterapia*.

[39] Cramer C., Proudfoot K., Ollivett T. (2020). Automated Feeding Behaviors Associated With Subclinical Respiratory Disease in Preweaned Dairy Calves. *Animals*.

[40] Buczinski S., Forté G., Bélanger A. M. (2013). Short Communication: Ultrasonographic Assessment of the Thorax as a Fast Technique to Assess Pulmonary Lesions in Dairy Calves With Bovine Respiratory Disease. *Journal of Dairy Science*.

[41] Shecaira C. D., Azedo M. R., Seino C. H. (2025). Dynamics of Immunity in Holstein Calves During the Neonatal Period: Evaluation of Leukogram, Cytokine Gene Expression, and T Lymphocytes. *Ciencia Animal Brasileira*.

[42] Kolar Q. K., Waddell L. A., Raper A. (2020). Anatomical Distribution of Respiratory Tract Leukocyte Cell Subsets in Neonatal Calves. *Veterinary Immunology and Immunopathology*.

[43] Hulbert L. E., Moisá S. J. (2016). Stress, Immunity, and the Management of Calves. *Journal of Dairy Science*.

[44] Ożarowski M., Karpiński T. M. (2023). The Effects of Propolis on Viral Respiratory Diseases. *Molecules*.

[45] Prado-Calixto O. P., Mizubuti I. Y., Ribeiro E. L. A. (2017). Ingestive Behavior and Blood Profile in Sheep Fed With Diets Containing Powdered Propolis Extract Additives.

[46] Ježek J. O., Nemec M. A., Starič J. O., Klinkon M. A. (2011). Age Related Changes and Reference Intervals of Haematological Variables in Dairy Calves. *Bulletin of the Veterinary Institute in Pulawy*.

[47] Ahmadi F., Mudroň P., Ivančová P. (2023). Effects of European Propolis Administration on the Growth Performance, Health Status, Selected Blood Biochemical and Immunological Variables of Pre-Weaning Calves.

